# Discordance Between Perceived Risk and Cardiovascular Health in Women of Reproductive Age

**DOI:** 10.1016/j.jacadv.2026.102862

**Published:** 2026-06-19

**Authors:** Ketum Ateh Stanislas, Faith E. Metlock, Asma Rayani, Ana Baez Mateo, Lilian Hernandez, Chioma Ezuma, Dhananjay Vaidya, Oluwabunmi Ogungbe, Pamela Ouyang, Melissa Hladek, Garima Sharma, Yvonne Commodore-Mensah

**Affiliations:** a Johns Hopkins School of Nursing, Baltimore, Maryland, USA; b Inova Heart and Vascular Institute, Fairfax, Virginia, USA; c Howard University College of Medicine, Washington, DC, USA

**Keywords:** cardiovascular health, discordance, Life's Essential 8, risk perception, women of reproductive age

## Abstract

**Background:**

Cardiovascular disease (CVD) remains the leading cause of death among women, yet awareness has declined significantly among women of reproductive age. Risk underestimation may impede prevention efforts during this critical life stage.

**Objectives:**

The objectives of the study were to evaluate concordance between perceived CVD and stroke risk and LE8-defined cardiovascular health status and to examine predictors of risk-cardiovascular health discordance among women of reproductive age.

**Methods:**

Cross-sectional study of 139 women aged 18 to 50 years in the Baltimore/Washington DC area. Participants assessed perceived CVD and stroke risk using 7-point Likert scales. Discordance was defined by cross-classifying perceived risk relative to peers with LE8-defined cardiovascular health status. Multivariable logistic regression examined predictors of underestimation.

**Results:**

Among 139 women (mean age 32.01 years), over half underestimated both CVD (56.8%) and stroke (58.3%) risk. In multivariable models, racial/ethnic minority status was significantly associated with underestimation of both CVD (OR: 3.70; 95% CI: 1.58-8.68) and stroke risk (OR: 3.29; 95% CI: 1.44-7.51), as was medium polysocial risk (CVD: OR: 2.72; 95% CI: 1.02-7.24; stroke: OR: 2.70; 95% CI: 1.04-7.02). Low cardiovascular health literacy (OR: 5.91; 95% CI: 1.05-33.31) and older reproductive age (≥32 years; OR: 2.26; 95% CI: 1.02-5.00) were additionally associated with CVD risk underestimation.

**Conclusions:**

CVD and stroke risk underestimation is prevalent among women of reproductive age with marked racial and social disparities. Targeted, culturally sensitive risk communication strategies are needed to improve CVD and stroke risk perception accuracy.

Cardiovascular disease (CVD) remains the leading cause of death for women in the United States, accounting for about 1 in every 3 female deaths.^1^ Despite this, a persistent myth has long defined how the public and even some in the medical community think about CVD; that it is primarily a “man’s problem”.^2^ Historically, women were under-represented in CVD research and clinical trials, limiting the generalizability of prevention and treatment evidence to women. In response, national efforts including the National Institutes of Health Office of Research on Women’s Health prioritized inclusion of women in biomedical research and accelerated sex-specific evidence generation.^3^ These broader movements, alongside public campaigns such as Go Red for Women,^4^ initially improved recognition of CVD risk among women; however, these gains were not sustained. By 2019, awareness of coronary heart disease as the top killer of women had declined to 44% down from 65% in 2009, with particularly low awareness among women aged 25 to 64 years.^5^ This decline, coupled with rising heart attack rates among midlife women,^6^ underscores an urgent need for better CVD prevention strategies for women in their reproductive years.

During the reproductive years, women experience distinct biological transitions and social demands that may shape how CVD risk^7^ is perceived and prioritized. One of the biggest hurdles to effective prevention appears to be a psychological phenomenon called “optimistic bias,” where women consistently underestimate their own personal risk.^8^ Many studies have shown that a significant number of women, even those with multiple objective risk factors, perceive their personal risk for CVD to be low or, at best, moderate.^9^ This is particularly concerning because a person's accurate understanding of their own risk is often essential for motivating behavior change and adherence to prevention plans.^10^^,^^11^ However, evidence suggests that a patient's risk perception relative to their peers might be a more powerful motivator for action than a simple numerical estimate of absolute risk.^12^ These insights lead to a critical question: what specific factors influence this perception among women of reproductive age, and why does this underestimation persist?

To address these knowledge gaps, this study had 3 primary objectives. First, we determined the concordance between a woman's perceived CVD and stroke risk, as measured by a comparative self-assessment scale,^13^ and her objective measure of cardiovascular health (CVH) metrics, the American Heart Association's (AHA) Life’s Essential 8 (LE8) score. The LE8 score has been prospectively validated against incident cardiovascular events, including stroke and atrial fibrillation, in multiple large cohorts.^14^, ^15^, ^16^ Second, we determined the demographic, clinical, and psychosocial predictors of CVD and stroke risk underestimation. Finally, we assessed whether the participants accurately perceived their risk relative to their peers. By quantifying the gap between a woman's subjective risk perception and her LE8 score, and by identifying the predictors of that discrepancy, our study provides insights that can help health educators create more effective and personalized CVH risk communication for women of reproductive age.

## Methods

### Study design and participants

This study utilized data from The Social Determinants of the Risk of Hypertension Study in Women of Reproductive Age (SAFE HEART) Study,^17^ a community-based cross-sectional study conducted in the Baltimore and Washington, DC, metropolitan areas. The overarching design of the SAFE HEART Study has been described in detail elsewhere^17^ and focused on examining social risk factors and CVH literacy (CVHL) among women of reproductive age. During SAFE HEART Study Phase II in 2024, we recruited 231 women from the Baltimore and Washington, DC, metropolitan area through community-based outreach efforts. Eligible participants provided consent and were between 18 and 50 years old, self-identified as women, and were able to speak and read English or Spanish.

The SAFE HEART study was reviewed and approved by the Johns Hopkins Medicine Institutional Review Board (IRB00337704), and all participants provided informed consent before participation.

### Measures

#### Perceived risk

Perceived risk of CVD and stroke was assessed using a 7-point comparative Likert scale adapted from prior risk perception studies.^18^ For CVD, participants were asked: “If I compare myself with others of my age and sex, I think my chance of having heart disease is:” with the following response options: much below average (−3); below average (−2); slightly below average (−1); average (0); slightly above average (+1); above average (+2); and much above average (+3). An identical format was used to assess perceived stroke risk.

For analysis, responses were recorded on a numeric scale from 1 to 7, corresponding to −3 through +3 respectively, with higher values indicating greater perceived risk. For analysis, perceived risk was dichotomized into low/average (≤4) vs elevated (>4) perceived risk.

#### Actual cardiovascular health and risk factors

CVH was assessed using the AHA LE8 score, which integrates 4 health behaviors (self-reported diet, physical activity, nicotine exposure, and sleep duration) and 4 health factors (body mass index, blood pressure, non–high-density lipoprotein cholesterol, and blood glucose or glycosylated hemoglobin were obtained through direct anthropometric, clinical, and laboratory measurement) on a 0 to 100 scale^19^ and has been prospectively associated with incident CVD and stroke.^14^

The overall LE8 score was categorized from low (0-49), moderate (50-79), or high (80-100) CVH. Low and moderate CVH categories were combined due to a relatively small proportion of participants in the low CVH group^20^ resulting in 2 groups: “suboptimal” (low or moderate CVH 0-79) and “ideal” (high CVH 80-100) CVH.^21^ The total number of self-reported cardiovascular risk factors (from the LE8 components) was also calculated for each participant and dichotomized into a low-risk group (0-1 risk factors) and an elevated-risk group (≥2 risk factors).^19^

#### Risk perception concordance classification

To assess concordance between objective CVH and subjective risk perception, we created a four-category risk perception concordance variable by cross-classifying LE8-based CVH status with perceived risk for CVD and stroke separately. Participants were classified as: 1) underestimation, those with suboptimal CVH (low/moderate CVH [LE8 < 80]) who perceived their risk as low or average (Likert score ≤4); 2) accurate-moderate/high risk, those with suboptimal CVH (low/moderate CVH [LE8 < 80]) who perceived their risk as elevated (Likert score >4); 3) accurate-low risk, those with ideal CVH (high CVH [LE8 ≥80]) who perceived their risk as low or average (Likert score ≤4); and 4) Overestimation, those with ideal CVH (high CVH [LE8 ≥80]) who perceived their risk as elevated (Likert score >4).

This classification scheme enabled identification of risk perception discordance, with particular focus on those who underestimated their risk as this group represents individuals at low or moderate CVH (LE8 <80) who may not engage in appropriate preventive behaviors.^19^

For kappa agreement analysis, perceived risk responses were divided into 3 risk groups of less (scores −3 to −1), same (score 0), or higher (scores +1 to +3) risk relative to peer based on age tertiles and the LE8-defined CVH status was similarly categorized into 3 risk groups by age tertiles. This ensured clinically interpretable risk perception categories and adequate cell sizes for analysis.

### Predictors of interest

Predictors were selected a priori and included age, race, polysocial risk score,^22^ and psychological factors (perceived stress and depressive symptoms) and CVHL. Age was modeled as a dichotomous variable using the sample median (<32 vs ≥32 years). This cutoff was selected to examine potential differences in cardiovascular risk perception between early and later reproductive adulthood and is supported by emerging evidence linking reproductive aging with accelerated cardiometabolic risk.^23^ Race and ethnicity were self-reported and categorized as non-Hispanic White, non-Hispanic Black/African American, non-Hispanic Asian, American Indian/Alaska Native, and other/multiracial. These were collapsed into a dichotomous variable (non-Hispanic White vs racial/ethnic minority) due to sample size constraints. Race/ethnicity is examined here as a social construct reflecting differential exposure to structural determinants of CVH, not as a biological variable. The polysocial risk score was a composite measure of 14 distinct social risk factors across 6 domains (socioeconomic stability, living situation, food security, transportation, utilities, and interpersonal safety) and was categorized into tertiles (low, medium, and high). High stress was defined using a dichotomized Perceived Stress Scale score (>8 in this sample), whereas depressive symptoms were defined using a dichotomized Patient Health Questionnaire score (≥3).^24^ CVHL was measured with the Heart Disease Facts questionnaire, which provides a score from 0 to 100 and used as binary variable with the threshold of 50 as the low cutoff, ≤70 for moderate, and >70 for high literacy.^25^

### Statistical analysis

Sample size was estimated using the events-per-variable rule,^26^ requiring ≥84 participants for 4 predictors at 60% expected outcome prevalence,^27^ a minimum of 84 participants (or 147 participants for 6 predictors) was estimated. Descriptive statistics are presented as frequencies and percentages for categorical variables and means ± SDS for continuous variables. To achieve the study’s objectives, the prevalence of each risk perception concordance category was calculated separately for CVD and stroke and presented in a horizontal bar graph. A kappa agreement test was used to compare how accurately the women perceived their LE8-defined CVH status relative to their peers of the same age tertile. Multivariable logistic regression models were used to determine predictors of perceived risk underestimation, including age, CVHL, polysocial risk, number of cardiovascular risk factors and stress and depression. Model 1 adjusting for demographic factors; model 2 adding polysocial risk; model 3 adding CVH risk factors; and models 4 to 5 adding psychological factors. Sensitivity analysis was used to select the model with best fit. All statistical analyses were performed using Stata (18.0; StataCorp).

## Results

### Participant characteristics

**Figure 1:**
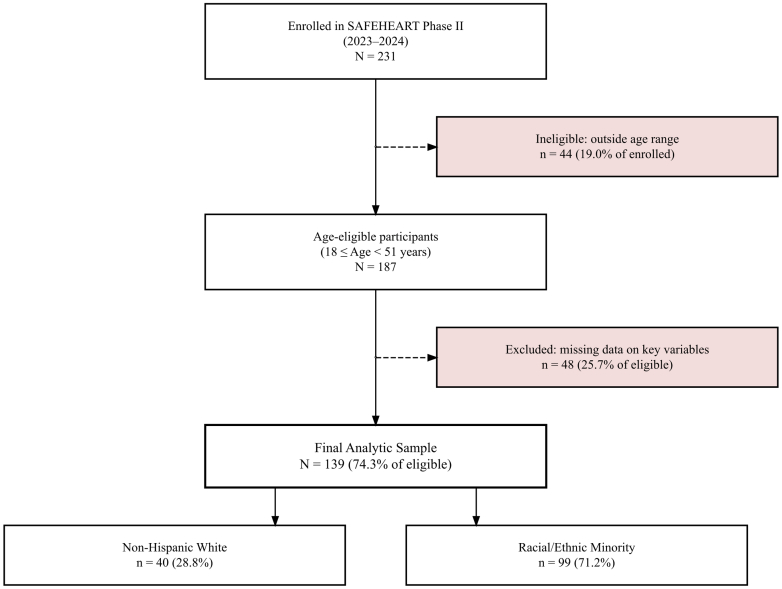
**Study Flow Diagram** This figure illustrates participant selection from the SAFE HEART Study Phase II. SAFE HEART = Social Determinants of the Risk of Hypertension Study in Women.

Of the 231 women initially enrolled, 187 were eligible. Of these, 139 had complete data on all key variables and were included in the primary analyses; 48 were excluded due to missing data (Figure 1). Excluded and included participants were comparable across most baseline characteristics, although excluded participants were more likely to identify as a racial or ethnic minority (88% vs 71%; *P* = 0.024) (Supplemental Table 1).

The cohort comprised women of reproductive age with a mean age of 32.0 ± 8.1 years, with slightly more than half aged 32 years or older (53%). Most participants identified as coming from racial/ethnic minority groups (71%). Overall CVH clustered primarily within the moderate range, with a mean LE8 score of 70.8 (SD 13.5). Among the 98 women classified as having low or moderate CVH (LE8 < 80), the median LE8 score was 66.25 (IQR: 58.75-73.75), indicating that most fell in the upper end of the moderate range (Table 1) Component-level distributions of LE8 scores (Supplemental Table 2) revealed that the largest proportions of women scoring in the low category were observed for body mass index (45.3%), diet (41.7%), and physical activity (36.0%). Most participants were classified as having suboptimal CVH (71%) and elevated CVD risk based on the presence of 2 or more CVD risk factors (83%).

**Table 1:** Demographic and Clinical Characteristics (N = 139)

Age, y, mean ± SD	32.01 ± (8.14)
Age	
<32 y	66 (47)
≥32 y	73 (53)
Race	
Non-Hispanic White	40 (29)
Racial/ethnic minority	99 (71)
Life's Essential 8 score	70.83 ± (13.52)
LE8 cardiovascular health category	
Low cardiovascular health	9 (6)
Moderate cardiovascular health	89 (64)
High cardiovascular health	41 (29)
CVH status	
Ideal CVH	41 (29)
Suboptimal CVH	98 (71)
Suboptimal CVH LE8 score, median (IQR)	66.25 (58.75-73.75)
Overall CVHL score (0-100)	76.00 (72.00-84.00)
CVHL category (HDFQ criteria)	
Good knowledge	109 (78)
Moderate knowledge	18 (13)
Low/poor knowledge	12 (9)
Polysocial risk	
Low	63 (45)
Medium	33 (24)
High	43 (31)
Number of CVH risk factors	
Low risk (0-1)	23 (17)
Elevated risk (2+)	116 (83)
Dichotomized PHQ-2 score (≥3 high)	
Low	122 (88)
High	17 (12)
Dichotomized PSS-4 score (≥8 high)	
Low	61 (44)
High	78 (56)

Values are mean ± SD, median (IQR), or n (%).

Cardiovascular health (CVH) categories follow the American Heart Association Life’s Essential 8 (LE8) tier definitions: low (0-49), moderate (50-79), and high (80-100). The binary CVH status (high vs low/moderate) was used in the primary concordance analysis; the three-tier distribution is reported for descriptive purposes per AHA convention.

CVHL = cardiovascular health literacy; HDFQ = Heart Disease Fact Questionnaire; PHQ-2 = Patient Health Questionnaire-2; PSS-4 = Perceived Stress Scale-4.

CVHL was generally high, with a median score of 76.0 (IQR: 72.0-84.0), although 22% demonstrated moderate or low knowledge. Psychosocial burden was common, with more than half reporting high perceived stress (56%) and 12% screening positive for depressive symptoms. Polysocial risk exposures were widely distributed, with 45% classified as low risk, 24% as medium risk, and 31% as high risk (Table 1).

### Risk perception accuracy assessment

**Figure 2:**
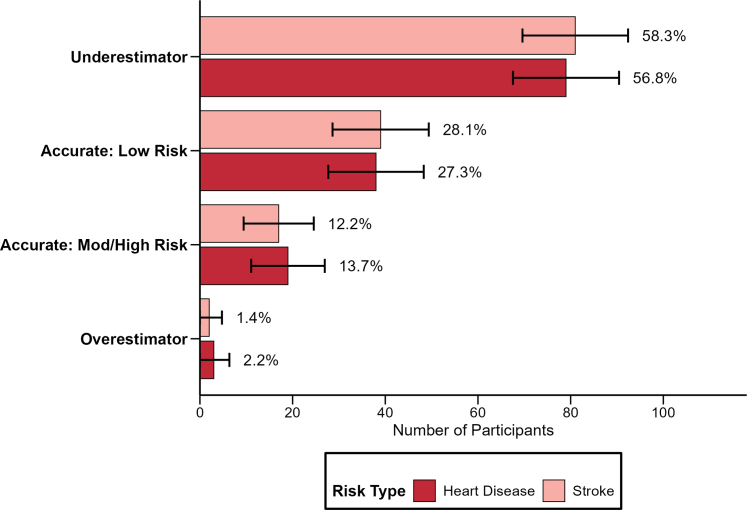
**Risk Perception Discordance for Cardiovascular Disease and Stroke** Distribution of risk perception accuracy categories, derived by cross-classifying perceived risk with Life's Essential 8 cardiovascular health status. Over half of participants underestimated both heart disease (56.8%) and stroke (58.3%) risk, whereas overestimation was rare (<3%). The similar pattern across both outcomes suggests shared mechanisms of discordance between perceived risk and objective CVH. Bar lengths represent participant counts; annotated values indicate percentages; error bars represent 95% CIs for the proportion of women in each category. Mod = moderate.

Risk perception concordance categories for CVD and stroke are shown in Figure 2.

For CVD risk, underestimation predominated, occurring in 56.8% of participants. Accurate risk perception was observed in 41.0%, including 27.3% who accurately perceived low risk and 13.7% who accurately perceived moderate to high risk. Risk overestimation was uncommon (2.2%).

For stroke risk, underestimation occurred in 58.3% of participants, accurate perception in 40.3% (28.1% low risk; 12.2% moderate to high risk) and overestimation in 1.4%.

### Agreement between perceived risk relative to peers and LE8-defined CVH

Perceived CVD risk relative to peers demonstrated poor concordance with objective age-specific LE8 tertile rankings for both outcomes. For CVD, observed agreement was 54.3%, which was lower than the agreement expected by chance alone (59.0%), yielding a weighted kappa coefficient of −0.11, indicating negligible agreement.

Similarly, for stroke risk, observed agreement was 55.6% compared with an expected agreement of 58.9%, with a weighted kappa of −0.08 (Supplemental Tables 3 and 4).

### Predictors of risk underestimation

#### Cardiovascular disease risk underestimation

**Figure 3:**
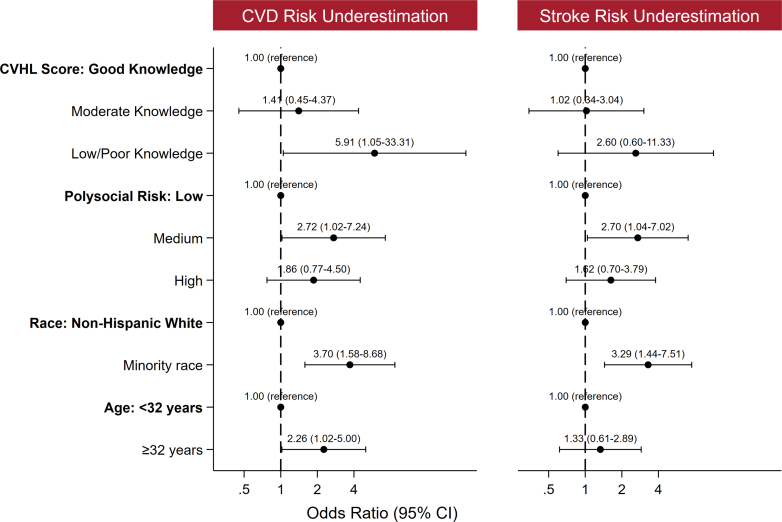
**Predictors of Cardiovascular Disease and Stroke Risk Underestimation Among Women of Reproductive Age** Multivariable logistic regression results identifying predictors of risk underestimation. Models included age, cardiovascular health literacy, polysocial risk, and race/ethnicity, selected by lowest Akaike information criterion (AUC = 0.75 for CVD; 0.71 for stroke; Hosmer-Lemeshow *P* = 0.453 and *P* = 0.310, respectively). Racial/ethnic minority status was the strongest predictor for both outcomes, suggesting structural factors in risk communication warrant attention. ORs are presented with 95% CIs. CVD = cardiovascular disease; CVHL = cardiovascular health literacy.

Multivariable logistic regression identified CVHL, polysocial risk, race/ethnicity, and age as key predictors of CVD risk underestimation (Figure 3, Supplemental Table 5). Participants with low or poor CVHL exhibited markedly higher odds of underestimating CVD risk compared with those with good knowledge (OR: 5.91; 95% CI: 1.05-33.31). Medium polysocial risk was associated with a significantly higher odds of underestimation (OR: 2.72; 95% CI: 1.02-7.24). Racial/ethnic minority status emerged as the strongest predictor across all models, with nearly four-fold higher odds of underestimation compared with non-Hispanic White participants (OR: 3.70; 95% CI: 1.58-8.68). Older reproductive age (≥32 years) was also associated with greater underestimation (OR: 2.26; 95% CI: 1.02-5.00).

Psychological distress measures, including perceived stress and depressive symptoms, were not independently associated with increased CVD risk underestimation.

#### Stroke risk underestimation

Medium polysocial risk remained significantly associated with higher odds of stroke risk underestimation (OR: 2.70; 95% CI: 1.04-7.02), whereas high polysocial risk demonstrated a similar but nonsignificant pattern. Racial/ethnic minority participants had more than three-fold higher odds of underestimating stroke risk compared with White participants (OR: 3.29; 95% CI: 1.44-7.51). Age and depressive symptoms were not significant predictors in stroke models. Elevated perceived stress was not significantly associated with stroke risk underestimation (Figure 3, Supplemental Table 6). In a sensitivity analysis using the AHA three-tier LE8 categorization (low, moderate, and high) with high CVH as the reference, the adjusted odds of perceiving CVD and stroke risk lower than peers were lower for women with moderate CVH (CVD: adjusted OR [aOR]: 0.48; 95% CI: 0.20-1.17; stroke: aOR: 0.40; 95% CI: 0.17-0.94) and low CVH (CVD: aOR: 0.39; 95% CI: 0.06-2.39; stroke: aOR: 0.21; 95% CI: 0.03-1.29) compared with high CVH women. Directional effects on non-LE8 predictors are consistent with primary findings, although statistical power is reduced. In a restricted analysis among women with LE8 <80 (n = 98), the odds of perceiving CVD risk lower than peers did not differ between low and moderate CVH women (aOR: 0.76; 95% CI: 0.14-4.20; *P* = 0.749), supporting the decision to collapse these categories in the primary analysis (Supplemental Table 7).

## Discussion

The study revealed substantial discordance between perceived risk and CVH status for both CVD and stroke with a high prevalence of underestimation of both CVD and stroke risk among women of reproductive age. Low CVHL, elevated polysocial risk, older age within reproductive years, and racial/ethnic minority emerged as significant predictors of CVD risk underestimation. Stroke risk underestimation was predicted by medium and high polysocial risk and racial/ethnic minority status. High depression scores were associated with lower odds of underestimating both CVD and stroke risk. Agreement between perceived and LE8-defined CVH relative to age-matched peers was poor for both outcomes, highlighting gaps in risk awareness that may undermine preventive care during a critical period for establishing long-term CVH in women (Central Illustration).^28^

**Central Illustration:**
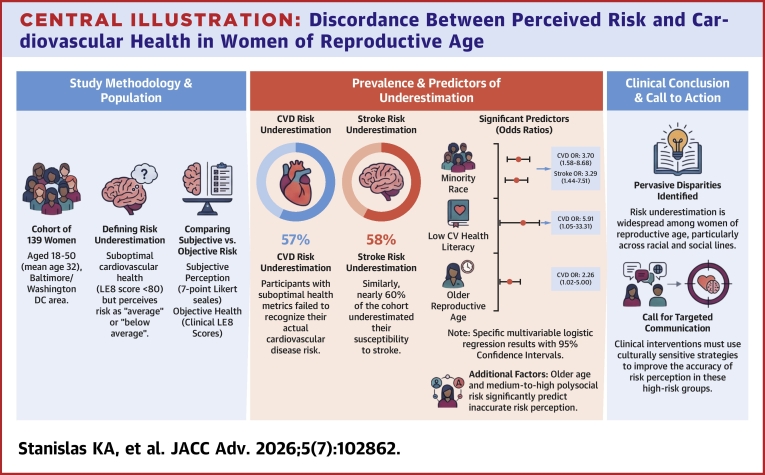
**Discordance Between Perceived Risk and Cardiovascular Health in Women of Reproductive Age** Among 139 women aged 18 to 50 years in the SAFE HEART Study (Baltimore/Washington, DC), perceived CVD and stroke risk were compared with objective cardiovascular health assessed using the American Heart Association Life's Essential 8 metrics. More than half of participants underestimated both CVD and stroke risk relative to their peers. Racial/ethnic minority status emerged as the strongest predictor of risk underestimation, with additional contributions from lower cardiovascular health literacy, higher polysocial risk, and older reproductive age. These findings highlight gaps in cardiovascular risk perception and suggest integrating health literacy assessment, social risk screening, and culturally sensitive risk communication into preventive care. CVD = cardiovascular disease; LE8 = Life's Essential 8.

Widespread underestimation of cardiovascular and stroke risk was observed among women of reproductive age, even among participants with multiple objective risk factors. This finding aligns with prior research demonstrating persistent misperception of cardiovascular risk among women.^29^ The observed misperception also parallels the documented decline in awareness of CVD as the leading cause of death among women over the past decade in the 2019 AHA National Survey.^5^ In that survey, the sharpest declines in awareness was most pronounced among Hispanic and non-Hispanic Black women and those with lower educational attainment, the same groups demonstrating the highest rates of risk underestimation in our cohort. Our findings extend this evidence by showing that population-level awareness gaps translate into individual-level discordance between perceived risk and objective CVH even after adjustment for objective CVH status. Inaccurate risk perception is clinically important because individuals who underestimate their risk are less likely to engage in preventive behaviors or adopt risk-reduction strategies.^10^^,^^30^ The similar patterns of underestimation across both cardiovascular and stroke outcomes suggest shared mechanisms underlying discordance between perceived risk and objective CVH rather than disease-specific knowledge gaps.

Poor CVHL was independently associated with underestimation of CVD risk, consistent with prior studies linking higher health literacy to more accurate cardiovascular risk perception.^31^ However, although previous research has linked health literacy to cardiovascular outcomes,^4^ the specific mechanisms through which health literacy shapes risk perception in younger women of reproductive age remains understudied. In addition, medium polysocial risk was consistently associated with greater risk underestimation, suggesting that moderate social stress may create cognitive burden without triggering the heightened health vigilance sometimes observed under more severe adversity. This pattern aligns with literature linking social strain to optimistic bias and reduced prioritization of preventive health behaviors.^32^ Notably, stroke risk underestimation followed a slightly different pattern with respect to health literacy, suggesting that cardiovascular and stroke risk may occupy different places in women’s health awareness, potentially reflecting differences in public health messaging and disease salience.^33^ Together, these findings suggest that both informational barriers and social context shape how women of reproductive age interpret their cardiovascular risk.

Participants from racial and ethnic minority populations were substantially more likely to underestimate both cardiovascular and stroke risk. These disparities persisted after adjustment for objective risk factors, health literacy, and psychosocial variables, suggesting the influence of broader structural determinants of health. Prior research has documented inequities in access to preventive cardiovascular care, biases embedded in risk communication tools,^34^ and differences in the quality of provider-patient communication that may affect how cardiovascular risk information is conveyed and understood.^35^^,^^36^ Structural factors such as experiences of discrimination, neighborhood context, and systemic barriers to care may therefore contribute to persistent disparities in cardiovascular risk perception among women.^37^^,^^38^

Additional findings provide further context for how women of reproductive age interpret cardiovascular risk. Older women of reproductive age demonstrated higher rates of CVD risk underestimation, a pattern consistent with prior research suggesting that competing midlife priorities may reduce attention to long-term cardiovascular risk.^39^ Women with multiple CVD risk factors were also more likely to underestimate their risk, consistent with prior work among urban Black women demonstrating substantial underestimation among those with multiple risk factors.^40^ Psychological factors showed variable associations with risk underestimation; although depressive symptoms were associated with lower odds of underestimation, the use of brief screening instruments (Patient Health Questionnaire-2 and Perceived Stress Scale-4), and the lack of statistical significance limit firm conclusions regarding these relationships. Prior work suggests that psychological processes such as denial,^18^ depressive realism,^41^ or differential disease awareness, may influence risk perception. Future studies using more comprehensive psychological assessments may clarify the role of psychological factors in cardiovascular risk perception. Reproductive-specific risk factors such as pregnancy complications and hormonal influences are also often absent from traditional cardiovascular risk communication frameworks, which may further contribute to inaccurate risk self-assessment among women during the reproductive years.^5^^,^^42^

Together, these findings suggest that current cardiovascular risk communication approaches may not adequately translate objective cardiovascular risk into patient understanding among women of reproductive age. Incorporating brief assessments of risk perception and CVHL into clinical encounters may help identify women who underestimate their risk and enable more targeted risk communication strategies. Clinicians may also consider contextualizing cardiovascular risk within age-specific peer comparisons and incorporating reproductive history into routine cardiovascular risk discussions. At a population level, tailored awareness initiatives addressing the life context of women of reproductive age may complement clinical strategies to improve cardiovascular risk awareness and preventive engagement.

Although LE8 was originally introduced as a cross-sectional CVH metric rather than a risk prediction tool, multiple recent prospective cohort studies have established robust associations between LE8 score and incident cardiovascular events. Wu et al.^14^ demonstrated that ideal vs poor CVH was significantly associated with a lower stroke risk in a community cohort and also using a meta-analysis of 11 studies. These findings have been replicated in the EPIC-NL Dutch cohort^15^ and in long-term LE8 trajectory analyses.^16^^,^^43^

### Study limitations

Several limitations should be considered. The study sample was drawn from English- and Spanish-speaking women in the Baltimore-Washington metropolitan area, which may limit generalizability to other regions, health care settings, or language groups. The LE8, although providing a comprehensive assessment of CVH, assigns equal weight to its components and does not incorporate reproductive risk factors, lipoprotein(a), and family history of CVD; our findings therefore reflect discordance between perceived risk and LE8-defined CVH rather than a comprehensive clinical risk estimate. Self-reported measures of risk perception, health literacy, and psychosocial factors may be subject to reporting bias. Furthermore, about 26% of eligible participants were excluded for missing data, and excluded participants were disproportionately racial/ethnic minorities; because this group reports lower cardiovascular risk awareness,^5^ our findings may underestimate discordance between perceived risk and objective CVH in more diverse populations. The modest sample size limited statistical precision precluded examination of predictor interactions, and required aggregation of diverse racial/ethnic groups, which may mask important within-group differences. Despite these limitations, this study addresses an important evidence gap. Although recent studies have characterized CVH prevalence in reproductive-age women using LE8^44^ and prior work has examined CVD risk perception using pregnancy history as the referent,^29^ comparison of perceived risk against LE8-defined CVH in this population remains largely unexplored. By identifying modifiable predictors of risk-CVH discordance, these findings may inform targeted strategies to improve cardiovascular risk communication during the reproductive years.

## Conclusions

Substantial risk-perception-CVH discordance and underestimation of cardiovascular and stroke risk was observed among women of reproductive age in this cohort. Racial and ethnic minority status, lower CVHL, and higher polysocial risk were associated with greater likelihood of risk underestimation. The discordance between perceived and objective cardiovascular risk relative to peers highlights important challenges in cardiovascular risk communication during the reproductive years. Improving CVHL and incorporating reproductive health context into risk communication strategies may help improve risk awareness and support more effective prevention among women of reproductive age.

Perspectives **COMPETENCY IN MEDICAL KNOWLEDGE:** This study evaluates concordance between perceived cardiovascular risk and LE8-defined CVH status in women of reproductive age, a relationship that remains largely unexplored in this population. In a community-based sample, concordance between perceived risk and actual CVH status was poor, with more than half of participants underestimating their risk relative to peers. Racial/ethnic minority status, lower CVHL, and higher polysocial risk emerged as key predictors of this risk underestimation, highlighting modifiable targets for intervention.

**TRANSLATIONAL OUTLOOK:** CVD prevention in women of reproductive age should incorporate structured risk communication that contextualizes CVH status relative to peers, particularly among women with lower health literacy and higher social risk burden. Integrating CVHL assessment and social risk screening into community and outpatient women’s health settings may help identify women whose perceived risk does not align with their CVH profile. Addressing disparities in risk perception–CVH concordance may support more equitable delivery of preventive interventions during the reproductive years.

## Funding support and author disclosures

The study was funded by 10.13039/100000968American Heart Association Grant Number 979462. The authors have reported that they have no relationships relevant to the contents of this paper to disclose.
