# Supplementary material for: Discordance Between Perceived Risk and Cardiovascular Health in Women of Reproductive Age
Source: JACC Adv. 2026 Jun 19;5(7):102862. doi: 10.1016/j.jacadv.2026.102862 (PMC13314748; doi:10.1016/j.jacadv.2026.102862)
Supplement: Supplemental_Material [file mmc1.pdf]

Supplementary Materials

Supplemental Table S1. Baseline characteristics of included versus excluded participants.

**Characteristic**	**Excluded (n = 48)**	**Included (n = 139)**	**Total (N = 187)**	**P-value**
Mean age, years	33.48 ± 8.22	32.01 ± 8.14	32.39 ± 8.17	0.327
**Age category, n (%)**				
<32 years	17 (35)	66 (47)	83 (44)	
≥32 years	31 (65)	73 (53)	104 (56)	0.147
Life's Essential 8 score, mean ± SD	60.42 ± 13.61	70.83 ± 13.52	70.61 ± 13.56	0.148
**AHA LE8 cardiovascular health category, n (%)**				
Low	1 (33)	9 (6)	10 (7)	
Moderate	2 (67)	89 (64)	91 (64)	
High	0 (0)	41 (29)	41 (29)	0.143
Missing, n	45	0	45	
**CVH status, n (%)**				
Optimal	0 (0)	41 (29)	41 (29)	
Suboptimal	3 (100)	98 (71)	101 (71)	0.265
Missing, n	45	0	45	
Overall CVHL score (0–100), median (IQR)	79.17 (56.52–100.00)	76.00 (72.00–84.00)	76.00 (70.71–84.31)	0.348
**CVHL category (HDFQ criteria), n (%)**				
Good knowledge	34 (71)	109 (78)	143 (76)	
Moderate knowledge	6 (12)	18 (13)	24 (13)	
Low/poor knowledge	8 (17)	12 (9)	20 (11)	0.297
**Polysocial risk, n (%)**				
Low	13 (27)	63 (45)	76 (41)	
Medium	16 (33)	33 (24)	49 (26)	
High	19 (40)	43 (31)	62 (33)	0.083
**Race/ethnicity, n (%)**				
Non-Hispanic White	6 (12)	40 (29)	46 (25)	
Racial/ethnic minority	42 (88)	99 (71)	141 (75)	**0.024**

Characteristic	Excluded (n = 48)	Included (n = 139)	Total (N = 187)	P-value
**Number of CVH risk factors, n (%)**				
Low risk (0–1)	8 (17)	23 (17)	31 (17)	
Elevated risk (2+)	40 (83)	116 (83)	156 (83)	0.985
**Dichotomized PHQ-2 score (≥3 high), n (%)**				
Low	44 (96)	122 (88)	166 (90)	
High	2 (4)	17 (12)	19 (10)	0.127
Missing, n	2	0	2	
**Dichotomized PSS-4 score (≥8 high), n (%)**				
Low	25 (54)	61 (44)	86 (46)	
High	21 (46)	78 (56)	99 (54)	0.217
Missing, n	2	0	2	

Notes.

Comparison of n = 139 included vs. n = 48 excluded participants. Participants were excluded due to missing key variables required for primary analyses.

Continuous variables: mean ± SD (or median [IQR] where indicated); p-values from t-test or Kruskal-Wallis test as appropriate.

Categorical variables: frequency (%); p-values from chi-square or Fisher's exact test. Percentages are calculated using participants with non-missing data as the denominator. Missing observations are reported beneath each variable where applicable.

Bold p-value indicates a statistically significant difference between groups ($p < 0.05$).

Abbreviations: AHA, American Heart Association; CVH, cardiovascular health; CVHL, cardiovascular health literacy; HDFQ, Heart Disease Fact Questionnaire; IQR, interquartile range; LE8, Life's Essential 8; PHQ-2, Patient Health Questionnaire-2; PSS-4, Perceived Stress Scale-4; SD, standard deviation.

Supplemental Table S2. Distribution of Life's Essential 8 component scores (N = 139).

LE8 Component	Low n (%)	Moderate n (%)	High n (%)	Mean ± SD
	Score denominator (100)			
**Health behaviors**				
Diet (DASH-style adherence)	58 (41.7)	42 (30.2)	39 (28.1)	43.6 ± 33.2
Physical activity	50 (36.0)	15 (10.8)	74 (53.2)	65.5 ± 36.0
Nicotine exposure	28 (20.1)	12 (8.6)	99 (71.2)	77.9 ± 38.3
Sleep duration	39 (28.1)	40 (28.8)	60 (43.2)	71.4 ± 29.5
**Health factors**				
Body mass index	63 (45.3)	40 (28.8)	36 (25.9)	53.8 ± 36.3
Blood pressure	5 (3.6)	35 (25.2)	99 (71.2)	86.2 ± 23.9
Blood lipids (non-HDL cholesterol)	22 (15.8)	22 (15.8)	95 (68.3)	80.7 ± 31.6
Blood glucose / HbA1c	18 (12.9)	15 (10.8)	106 (76.3)	87.6 ± 23.1
**Composite LE8 score**	**9 (6.5)**	**89 (64.0)**	**41 (29.5)**	**70.8 ± 13.5**

Notes.

Each Life's Essential 8 (LE8) component is scored 0-100 per American Heart Association guidance and categorized as Low (0-49), Moderate (50-79), or High (80-100). Higher scores indicate more favorable cardiovascular health.

Health behaviors (diet, physical activity, nicotine exposure, sleep duration) were assessed by self-report. Health factors (body mass index, blood pressure, non-HDL cholesterol, blood glucose or HbA1c) were obtained through direct anthropometric, clinical, and laboratory measurement.

The composite LE8 score is the unweighted mean of the eight component scores and is similarly categorized as Low, Moderate, or High.

Among participants with composite LE8 score < 80 (Low or Moderate CVH; n = 98), the median (IQR) score was 66.3 (58.8–73.8).

Percentages are calculated using N = 139 as the denominator; no missing observations were present for any component in the analytic sample. Row percentages may not sum to exactly 100 due to rounding.

Abbreviations: AHA, American Heart Association; DASH, Dietary Approaches to Stop Hypertension; HbA1c, glycated hemoglobin; HDL, high-density lipoprotein; SD, Standard Deviation; LE8, Life's Essential 8.

Supplementary Table S3. Actual-age-specific risk tertiles by perceived CVD risk categories

Perceived CVH risk category N (%)				
**Age-tertile**	**Less**	**Same**	**Higher**	**Total**
1st	13 (27.1)	22 (45.8)	13 (27.1)	48(100.0)
2nd	15(31.9)	26(55.3)	6(12.8)	47(100.0)
3rd	17(38.6)	24(54.6)	3(6.8)	44(100.0)
Total	45(32.4)	72(51.8)	22(15.8)	139
Statistics	$\kappa = -0.11$, SE = 0.06, $p = 0.97$			

Distribution of participants' objective cardiovascular health (CVH) tertiles (based on age-adjusted Life's Essential 8 scores) stratified by self-perceived CVD risk relative to same-age peers. Perceived risk categories collapsed into lower, same, and higher risk than peers. Data are presented as n (%); κ = weighted kappa statistic for agreement between actual and perceived risk. SE = standard error

**Supplementary Table S4. Actual-age-specific CVH risk tertiles by perceived stroke risk categories**				
		**Perceived stroke risk category N (%)**		
**Age-tertile**	**Less**	**Same**	**Higher**	**Total**
1st	14 (31.1)	21 (46.67)	10 (22.2)	45(100.0)
2nd	15(33.3)	24(53.3)	6(13.3)	45(100.0)
3rd	16(37.2)	24(55.8)	3(7.0)	43(100.0)
**Total**	45(33.8)	69(51.9)	19(14.3)	139
**statistic**	Weighted κ = -0.08, SE = 0.06, p = 0.90			

Distribution of participants' objective cardiovascular health (CVH) tertiles (based on age-adjusted Life's Essential 8 scores) stratified by self-perceived stroke risk relative to same-age peers. Perceived risk categories collapsed into lower, same, and higher risk than peers. Data are presented as n (%); κ = weighted kappa statistic for agreement between actual and perceived risk. SE = standard error

Supplementary Table S5. Predictors of Heart Risk Underestimation						
	**Univariate**	**Model 1**	**Model 2**	**Model 3**	**Model 4**	**Model 5**
**Predictor**	OR (95% CI)					
**Age**						
Age <32 years	1.00	1.00	1.00	1.00	1.00	1.00
Age ≥32 years	2.45 (1.23,4.87)	1.97 (0.92,4.20)	2.26 (1.02,5.00)	2.23 (0.99,4.98)	2.28 (1.01,5.14)	2.27 (1.01,5.14)
**Race[a]**						
Non-Hispanic White	1.00	1.00	1.00	1.00	1.00	1.00
Racial/ethnic minority	4.15 (1.90,9.08)	3.62 (1.58,8.31)	3.70 (1.58,8.68)	3.70 (1.58,8.68)	3.70 (1.58,8.67)	3.70 (1.58,8.67)
**CVHL**						
Good Knowledge	1.00	1.00	1.00	1.00	1.00	1.00
Moderate Knowledge	1.38 (0.50,3.83)	1.41 (0.45,4.37)	1.39 (0.44,4.34)	1.37 (0.44,4.30)	1.38 (0.44,4.35)	1.33 (0.44,3.98)
Low/Poor Knowledge	4.40 (0.92,21.01)	5.91 (1.05,33.31)	5.91 (1.05,33.32)	5.77 (1.02,32.60)	5.75 (1.02,32.58)	5.49 (1.04,29.02)
**Polysocial risk**						
Low	1.00		1.00	1.00	1.00	1.00
Moderate	1.69 (0.72,3.98)		2.72 (1.02,7.24)	2.70 (1.01,7.19)	2.78 (1.03,7.46)	2.77 (1.03,7.46)
High	2.28 (1.02,5.11)		1.86 (0.77,4.50)	1.85 (0.76,4.49)	1.97 (0.78,4.95)	1.97 (0.78,4.95)
**Risk Factors[b]**						
Low Risk (0-1)	1.00			1.00	1.00	1.00
Elevated Risk (2+)	1.55 (0.63,3.79)			1.12 (0.42,3.02)	1.12 (0.41,3.02)	1.12 (0.41,3.03)
**PHQ-2**						
low	1.00				1.00	1.00
high	1.10 (0.39,3.07)				0.75 (0.24,2.39)	0.76 (0.24,2.40)
**PSS-4**						
low	1.00					1.00
high	0.85 (0.43,1.68)					0.98 (0.45,2.10)
**AIC**		178.54	177.73	179.68	181.45	183.44
**AUC**		0.720675	0.750527	0.753481	0.750105	0.74884

Logistic regression models show odds ratios and 95% confidence intervals for CVD risk underestimation (N = 139). Logistic regression models show odds ratios and 95% confidence

intervals for CVD risk underestimation (N = 139). Models are sequentially adjusted, each building on the previous: Univariate (unadjusted), Model 1 (+ age, race, CVHL), Model 2 (+ polysocial risk), Model 3 (+ CVH risk factors), Model 4 (+ PHQ-2), Model 5 (+ PSS-4).

[a] Race: Non-Hispanic White (reference) vs. racial/ethnic minority (non-Hispanic Black, Hispanic, non-Hispanic Asian, and other/multiracial). [b] Risk factors: diabetes, high cholesterol, high blood pressure, smoked at least 100 cigarettes in lifetime, <150 minutes of physical activity, <5 servings of fruits and vegetables, not having 7–9 hours of sleep, BMI ≥25.

AIC = Akaike information criterion; AUC = area under the curve; BMI = body mass index; CI = confidence interval; CVD = cardiovascular disease; CVH = cardiovascular health; CVHL = cardiovascular health literacy; OR = odds ratio; PHQ-2 = Patient Health Questionnaire-2; PSS-4 = Perceived Stress Scale-4.

Supplementary Table S6. Predictors of Stroke Risk Underestimation						
	**Univariate**	**Model 1**	**Model 2**	**Model 3**	**Model 4**	**Model 5**
	OR (95% CI)					
**Age**						
Age <32 years	1.00	1.00	1.00	1.00	1.00	1.00
Age ≥32 years	1.51 (0.77,2.97)	1.17 (0.56-2.47)	1.33 (0.61-2.89)	1.26 (0.57-2.78)	1.29 (0.58-2.85)	1.31 (0.59-2.91)
**Race**						
Non-Hispanic White	1.00	1.00	1.00	1.00	1.00	1.00
Racial/ethnic minority	3.33 (1.55,7.16)	3.17 (1.42-7.08)	3.29 (1.44-7.51)	3.29 (1.44-7.54)	3.30 (1.44-7.56)	3.33 (1.45-7.66)
**CVHL**						
Good Knowledge	1.00	1.00	1.00	1.00	1.00	1.00
Moderate Knowledge	0.95 (0.35,2.59)	0.97 (0.34-2.79)	1.02 (0.34-3.04)	0.98 (0.33-2.94)	0.97 (0.32-2.92)	0.94 (0.31-2.85)
Low/Poor Knowledge	2.27 (0.58,8.87)	2.35 (0.57-9.66)	2.60 (0.60-11.33)	2.59 (0.59-11.34)	2.54 (0.58-11.16)	2.62 (0.59-11.62)
**Polysocial risk**						
Low	1.00		1.00	1.00	1.00	1.00
Moderate	2.06 (0.86,4.96)		2.70 (1.04-7.02)	2.63 (1.01-6.82)	2.69 (1.03-7.05)	2.74 (1.04-7.20)
High	1.93 (0.87,4.28)		1.62 (0.70-3.79)	1.60 (0.68-3.74)	1.68 (0.69-4.07)	1.69 (0.70-4.09)
**Risk Factors**						
Low Risk (0-1)	1.00			1.00	1.00	1.00
Elevated Risk (2+)	1.66 (0.68,4.08)			1.44 (0.54-3.83)	1.44 (0.54-3.82)	1.41 (0.53-3.77)
**PHQ-2**						
low	1.00				1.00	1.00
high	1.03 (0.37,2.88)				0.79 (0.26-2.43)	0.76 (0.24-2.37)
**PSS-4**						
low	1.00					1.00
high	1.07 (0.54,2.11)					1.24 (0.59-2.60)
**AIC**		187.27	186.64	188.11	189.93	191.62
**AUC**		0.65794	0.712218	0.716369	0.716582	0.713069

Supplementary Table 5. Logistic regression models show odds ratios and 95% confidence intervals for Stroke risk underestimation (N = 139). Logistic regression models show odds ratios and 95% confidence intervals for CVD risk underestimation (N = 139). Models are sequentially adjusted, each building on the previous: Univariate (unadjusted), Model 1 (+ age, race, CVHL), Model 2 (+ polysocial risk), Model 3 (+ CVH risk factors), Model 4 (+ PHQ-2), Model 5 (+ PSS-4).

[a] Race: Non-Hispanic White (reference) vs. racial/ethnic minority (non-Hispanic Black, Hispanic, non-Hispanic Asian, and other/multiracial). [b] Risk factors: diabetes, high cholesterol, high blood pressure, smoked at least 100 cigarettes in lifetime, <150 minutes of physical activity, <5 servings of fruits and vegetables, not having 7-9 hours of sleep, BMI ≥25.

AIC = Akaike information criterion; AUC = area under the curve; BMI = body mass index; CI = confidence interval; CVD = cardiovascular disease; CVH = cardiovascular health; CVHL = cardiovascular health literacy; OR = odds ratio; PHQ-2 = Patient Health Questionnaire-2; PSS-4 = Perceived Stress Scale-4.

Supplemental Table S7. Sensitivity analysis: adjusted odds of perceiving cardiovascular disease and stroke risk lower than peers, using three-tier Life's Essential 8 categorization (N = 139).

	**CVD risk perception lower than peers**		**Stroke risk perception lower than peers**	
**Predictor**	**aOR (95% CI)**	**P-value**	**aOR (95% CI)**	**P-value**
**Cardiovascular health category (LE8)**				
High CVH (≥80) [reference]	1.00 (ref)	—	1.00 (ref)	—
Moderate CVH (50–79)	0.48 (0.20–1.17)	0.107	0.40 (0.17–0.94)	0.037
Low CVH (0–49)	0.39 (0.06–2.39)	0.307	0.21 (0.03–1.29)	0.092
**Cardiovascular health literacy**				
Good knowledge [reference]	1.00 (ref)	—	1.00 (ref)	—
Moderate knowledge	0.78 (0.25–2.48)	0.677	0.61 (0.20–1.85)	0.380
Low/poor knowledge	2.87 (0.76–10.77)	0.119	1.36 (0.36–5.10)	0.651
**Polysocial risk**				
Low [reference]	1.00 (ref)	—	1.00 (ref)	—
Medium	0.64 (0.23–1.73)	0.376	0.80 (0.32–2.01)	0.635
High	0.59 (0.24–1.46)	0.254	0.49 (0.20–1.18)	0.114
**Race/ethnicity**				
Non-Hispanic White [reference]	1.00 (ref)	—	1.00 (ref)	—
Racial/ethnic minority	1.76 (0.71–4.40)	0.225	1.90 (0.79–4.56)	0.153

Predictor	CVD risk perception lower than peers aOR (95% CI)	CVD risk perception lower than peers P-value	Stroke risk perception lower than peers aOR (95% CI)	Stroke risk perception lower than peers P-value
**Age**				
<32 years [reference]	1.00 (ref)	—	1.00 (ref)	—
≥32 years	1.09 (0.49–2.42)	0.836	1.06 (0.49–2.28)	0.886

Notes.

Outcome: perceived risk lower than peers (Likert ≤ 3) versus same as or higher than peers (Likert ≥ 4).

Adjusted odds ratios (aOR) and 95% confidence intervals (CI) are from multivariable logistic regression mutually adjusting for all predictors shown. Cardiovascular health was operationalized using the American Heart Association Life's Essential 8 (LE8) three-tier categorization, with High CVH (LE8 ≥ 80) as the reference.

This sensitivity analysis complements the primary analysis (main manuscript Figure X and Table X), in which the binary underestimation outcome (objective LE8 < 80 and perceived risk at or below average) was regressed on a dichotomized CVH variable. The present specification uses perceived risk direction as the outcome and retains three-tier CVH as the predictor to align with AHA reporting convention.

In an additional restricted-sample sensitivity analysis limited to women with LE8 < 80 (n = 98), the adjusted odds of perceiving CVD risk lower than peers did not differ significantly between Low and Moderate CVH women (aOR = 0.76; 95% CI: 0.14–4.20; p = 0.749), supporting the operational decision to collapse Low and Moderate CVH in the primary analysis.

Abbreviations: AHA, American Heart Association; aOR, adjusted odds ratio; CI, confidence interval; CVD, cardiovascular disease; CVH, cardiovascular health; LE8, Life's Essential 8; ref, reference category.
